# Point specificity in acupuncture

**DOI:** 10.1186/1749-8546-7-4

**Published:** 2012-02-28

**Authors:** Emma M Choi, Fang Jiang, John C Longhurst

**Affiliations:** 1 Susan Samueli Center for Integrative Medicine, Department of Medicine, School of Medicine, University of California, Irvine CA 92697-4075, USA; 2 Medical Science 1C240, School of Medicine, University of California, Irvine CA 92697-4075, USA

## Abstract

The existence of point specificity in acupuncture is controversial, because many acupuncture studies using this principle to select control points have found that sham acupoints have similar effects to those of verum acupoints. Furthermore, the results of pain-related studies based on visual analogue scales have not supported the concept of point specificity. In contrast, hemodynamic, functional magnetic resonance imaging and neurophysiological studies evaluating the responses to stimulation of multiple points on the body surface have shown that point-specific actions are present. This review article focuses on clinical and laboratory studies supporting the existence of point specificity in acupuncture and also addresses studies that do not support this concept. Further research is needed to elucidate the point-specific actions of acupuncture.

## Background

Point specificity remains an important underlying principle used for prescribing acupuncture treatment in traditional Chinese medicine (TCM). According to TCM theory, stimulation of acupoints elicits functional responses that can be used to treat diseases [1]. Practitioners believe that the therapeutic effects of acupoint stimulation primarily work through 12 principal meridians that represent channels through which energy known as *Qi *flows [2]. Abnormal flow of *Qi *in meridians is related to disease in principal Chinese organs [3], and its natural flow is restored by stimulation of the appropriate acupoints [4]. When choosing the acupoints, acupuncturists first examine the patient's symptoms to determine which meridians are involved and subsequently choose a set of acupoints to stimulate along the meridians to evoke the greatest clinical responses [5]. For example, frontal headaches are treated by stimulating acupoints *Shangxing *(GV23) and *Hegu *(LI4), while occipital headaches are treated with acupoints *Fengchi *(GB20), *Kunlun *(B60) and *Lieque *(L7) [6]. Patients also are examined for the presence of *Ashi *points, which are nonspecific pressure points that elicit pain upon palpation and can be used along with the other acupoints to treat local pain [4]. Different acupoints may be stimulated at similar or different frequencies and intensities depending on the patient's conditions [1].

As TCM has been disseminated worldwide, different versions of acupuncture techniques have been developed, some of which do not follow the TCM principles for acupoint selection. For instance, 'trigger point acupuncture' involves stimulation of only *Ashi *points, single-point acupuncture uses only one acupoint for treatment and the eclectic approach involves needling anywhere within the region of the pain without any reference to *Ashi *or traditional acupoints [3]. Despite the various types of new acupuncture approaches, traditional point-specific techniques remain the most common form of acupuncture.

This article reviews clinical and laboratory studies on the role of point specificity. The aims of this article are not only to discuss acupuncture techniques, but also to evaluate the validity of the proposed mechanisms. The article's focus is on studies that incorporated sham controls with traditional needling techniques at non-acupoints or inactive acupoints to evaluate the principle of point specificity. The inclusion criteria take into account sample size and blinding of participants, location of the sham acupoints, needling technique and rationale for choosing both verum and sham acupoints.

## Point specificity in sham acupuncture controls

Because the mechanisms underlying the clinical and physiological responses to acupuncture remain unclear, various types of controls have been implemented in acupuncture research. For the purposes of this review, any type of placebo control for acupuncture has been classified as sham acupuncture. Many recent studies have used a non-penetrating sham control, referred to as the placebo needle [7-9]. Other placebo controls use minimal acupuncture and stimulation at inactive acupoints or non-acupoints involving skin penetration.

Minimal acupuncture utilizes needles inserted only 1-2 mm below the surface of the skin [10]. Needles are applied with no stimulation or light stimulation either mechanically by twirling the needles or electrically by applying a small pulsating current to the needles [10-14]. Despite the popular use of minimal acupuncture as a sham control, many clinical trials have suggested that this type of control has the potential to evoke analgesia through stimulation of cutaneous afferent nerves [15]. However, a recent study in mice reported that although superficial needle insertion caused analgesic effects, the effect was local and distinct from the acupuncture responses that rely on the afferent innervation of the skin [16]. In addition, the effects of electroacupuncture (EA) on blood pressure have been shown to result from stimulation of deeper nerves such as the median nerve located below *Jianshi-Neiguan *(P5-6) acupoints rather than from stimulation of superficial (i.e., cutaneous) branches such as the superficial radial nerve at *Panli-Wenliu *(LI6-LI7) acupoints [17,18]. In fact, investigation of reflex-induced pressor responses in cats showed that minimal or superficial acupuncture can serve as a valid control, because it does not alter the cardiovascular responses [19].

Needling at either inactive acupoints or non-acupoints are also common controls used in studies of acupuncture [20-24]. Non-acupoints are points located several millimeters or centimeters from verum acupoints, halfway between two parallel meridians or arbitrarily on the side of the trunk or the shoulder region away from most meridians [25]. Non-*Ashi *and non-acupoints are thought to have no therapeutic influence and thus may serve as inert placebo controls. Stimulation of inactive acupoints also is believed to be ineffective, so they can be used as sham controls. Inactive acupuncture points are chosen according to their anatomical locations, underlying neural pathways, corresponding Chinese meridians, proximity to verum acupoints and role in treating diseases [18,19,21,23]. With adequate blinding, the effects of stimulating either inactive acupoints or non-acupoints have been hypothesized to represent placebo responses.

The difference between sham and verum acupuncture remains unclear. Recent systematic reviews have suggested that stimulation of non-acupoints and inactive acupoints can elicit effects similar to those of stimulation of verum acupoints [26,27]. Thus, Langevin et al. [5] noted similar responses in sham and verum acupoint stimulation and attributed the nonspecific needling effects to either the summation of stimulation of multiple superficial sensory nerve endings or to the therapeutic effect related to practitioner-patient interactions. Despite these findings, some experts believe that it is illogical to conclude that stimulating all points on the body will elicit the same effect, because there is an uneven distribution of nerve endings throughout the body, suggesting differential sensory input at different points on the body [25]. In fact, many well-controlled studies do support the principle of point specificity. For example, recent studies in cardiovascular disease have shown that stimulation of verum acupoints elicits significantly greater responses than stimulation of both non-acupoints and inactive acupoints [18,19,23]. In addition, brain imaging studies have documented differential patterns of activation resulting from stimulation of inactive acupoints and non-acupoints [21,28]. Furthermore, experimental investigations have found that stimulation of different acupoints produces differential input to regions of the brain that regulate sympathetic outflow and cardiovascular function [18].

Because of contradictory results from many studies, some investigators have questioned the use of sham acupuncture as a placebo control [29,30]. Furthermore, some reviews argue that the principle of point specificity would be invalidated if stimulation at non-acupuncture points and inactive acupuncture points produced the same effects as verum acupuncture [26,27]. However, because the studies have used a wide variety of acupuncture techniques, controls and measureable outcomes, it is difficult to assess the validity of point specificity by evaluating this literature.

## Pain studies on point-specific responses

The majority of studies refuting the principle of point specificity evaluated the role of acupuncture in pain relief. Assefi et al. [31], for example, studied the actions of acupuncture at traditional acupoints and non-acupoints in 114 subjects diagnosed with fibromyalgia. They found that 25-35% of subjects experienced significant decreases in pain, regardless of the location of acupoint stimulation. Other acupuncture studies of fibromyalgia have reported similar results [32,33]. Because acupuncture frequently involves stimulation of *Ashi *points, which lie on painful points of the body without relation to meridians and acupoints, it is reasonable to speculate that non-acupoints used in fibromyalgia studies often are *Ashi *points [31]. Furthermore, acupuncture may have regional effects through the local release of neuromodulators such as adenosine [16]. Studies of fibromyalgia may not be able to accurately assess point specificity, because this condition frequently involves diffuse pain located in several regions of the body. Hence, it may be difficult in fibromyalgia to distinguish changes in pain threshold during stimulation of non-acupoints vs. verum acupoints.

Similarly, other studies of acupuncture in acute pain have not been able to validate the concept of point specificity [34-37]. For example, Linde et al. [34] reported that measurement of headache intensity after stimulation of verum acupoints and non-acupoints in 208 subjects showed no difference in the effectiveness of pain reduction by either method. These studies were generally well controlled with adequate numbers of subjects, but the outcomes were measured by a visual analogue scale (VAS), which is highly variable between individuals [38]. Thus, any differences between placebo and nonspecific actions between sham and verum acupuncture may not have been detected.

In contrast to most pain studies that utilize a VAS to measure pain, Cahn et al. [20] used a survey with yes or no answers to study acupuncture in pain in the pharynx and stomach after gastroscopy. Ninety subjects received either stimulation at 10 verum acupoints, including *Chengjiang *(CV24), *Lianquan *(CV23), *Shanzhong *(CV17), *Zhongwan *(CV12), *Shangqiu *(SP5), *Neiguan *(P6) and two at *Zusanli *(S36), or sham acupoints 1 cm from the verum acupoints. The researchers found that significantly fewer subjects in the verum acupuncture group experienced pain following treatment compared with the control group. These findings suggest that the inconclusive results in some studies of point specificity in pain could be due to variability in the measurement of outcomes, for example, using a VAS rather than a simpler approach of asking survey questions that have a bidirectional response.

## Hemodynamic studies on point-specific responses

Hemodynamic studies employ more objective and precise methods to evaluate acupoint and non-acupoint stimulation by precisely measuring heart rate and blood pressure. In a study comparing verum and non-acupoint effects, Huang et al. [22] assessed heart rate variability (HRV) in 111 subjects after stimulation of acupoint P6 and a sham acupoint located 1 cm to the ulnar side of P6. They showed that acupoint P6 stimulation decreased heart rate and increased the high-frequency HRV index of cardiac vagal modulation, while stimulation of the sham acupoint decreased heart rate without changing vagal outflow.

Other hemodynamic studies also have compared the responses to stimulation of inactive acupoints with those of verum acupuncture stimulation. In this regard, Li et al. [23] examined systolic, diastolic and mean blood pressure, heart rate and the rate-pressure product (RPP) as an index of myocardial oxygen demand, in 17 subjects before and after exercise. The researchers measured the exercise-induced pressor responses immediately following EA stimulation of P5-6, LI4-L7 and *Guangming-Xuanzhong *(GB37-39) acupoints. They found that EA applied at acupoints P5-6 and LI4-L7 increased the maximum workload and reduced systolic and mean blood pressures as well as the RPP during exercise, while EA applied to GB37-39 did not alter these hemodynamic responses. They concluded that stimulation of inactive acupoints does not alter the sympathoexcitatory responses to exercise as seen with stimulation of verum acupoints.

Similar observations have been made in experimental studies of the responses to stimulation of LI6-7, P5-6, *Yinxi-Shenmen *(H6-7) and *Zusanli-Shangjuxu *(S36-37) acupoints on the pressor responses from gastric distension in rats, a model used for investigating visceral excitatory cardiovascular reflexes [19]. The control acupoints LI6-7 were chosen since they have not been used to treat cardiovascular disease and have little input to regions of the brain that regulate cardiovascular function [18]. The results showed that EA at LI6-7 did not modulate the reflex hemodynamic responses, while stimulation at P5-6, H6-7 and S36-37 significantly reduced the sympathoexcitatory responses. Thus, while multiple acupoints can be used to modulate cardiovascular function, the responses to EA are quite point-specific.

In a comprehensive laboratory study of point specificity, Tjen-A-Looi et al. [18] evaluated changes in reflex increases in blood pressure caused by chemical stimulation of the gallbladder in anesthetized cats during individual stimulation of several sets of acupoints: P5-6, *Shousanli-Quchi *(LI10-11), LI4-L7, S36-37, LI6-7 and *Yongquan-Zhiyin *(K1-B67). P5-6, LI4-7, LI10-11 and S36-37 are commonly used to treat cardiovascular disease [18], while LI6-7 and K1-B67 are located along superficial nerves and were used as controls to test the difference between stimulating cutaneous and deep nerves. The investigators found that low-frequency EA stimulation of LI6-7 and K1-B67 did not influence the pressor response, while stimulation of the other sets of acupoints significantly reduced the reflex increases in blood pressure. Furthermore, P5-6 or LI10-11 modulated the response to a significantly greater extent and for a longer duration than LI4-L7 and S36-37. Thus, even though stimulation of many different sets of acupoints may be used to treat cardiovascular disease, their therapeutic effects, with respect to lowering of elevated blood pressure, are distinct in terms of magnitude and duration.

## Neurological studies on point-specific responses

One of the most important mechanisms underlying acupuncture's therapeutic effects is its action on the central nervous system [2]. With the development of non-invasive techniques such as functional magnetic resonance imaging (fMRI), which visualizes changes in blood flow and brain activity, studies on the neurological effects of acupuncture have become more practical in clinical settings. Several studies have compared changes in brain activity during stimulation of verum and sham acupoints to evaluate placebo responses and other factors (e.g., pain and nervousness), all of which commonly occur with both sham and verum acupuncture. For example, comparing verum and inactive acupoint stimulation in an fMRI study, Yoo et al. [39] found that stimulation of P6 and an inactive acupoint in 12 subjects activated a number of similar regions. However, stimulation of the inactive acupoints did not alter signals in the left precentral gyrus, dorsomedial nucleus of the thalamus, superior frontal gyrus or several neuro-matrices in the cerebellum, which were activated by stimulating P6. Notably, P6 has been used commonly to treat nausea [40], which is associated with cerebellar activity [41]. The cerebellum is activated during P6 stimulation, but not by sham stimulation, supporting the conclusion that acupoints are disease-specific.

Stimulating different verum acupoints also has been shown to elicit differential patterns of brain activity. Zhang et al. [42] for example found that stimulating different sets of acupoints to treat different conditions elicited distinct patterns of activation in the brain. This study compared stimulation of acupoints S36 and *Sanyinjiao *(SP6), used to treat visceral disorders [43], to *Yanglingquan *(GB34) and *Chengshan *(B57), used for disorders of the muscles and tendons [44]. The fMRI data showed that stimulation of S36 and SP6 activated the orbital frontal cortex and deactivated the hippocampus and parietal BA7, regions associated with visceral disorders [45,46]. On the other hand, stimulation of GB34 and B57 increased activity in the dorsal thalamus and inhibited activity in the primary motor and premotor cortices. Interestingly, even though the four acupoints are located within the same spinal segment, they elicited different responses when stimulated. These results suggest that stimulation of different sets of acupoints leads to disease-specific neuronal responses, even when acupoints are located within the same spinal segment.

A study [18] of the neural mechanisms underlying the effect of EA on cardiovascular diseases showed that the point-specific activation patterns were the result of stimulation of different somatic nerves that underlie meridians and acupoints. Moreover, studies have shown that stimulating acupoints along the median and peroneal nerves reduces hypertension by modulating activity in the rostral ventrolateral medulla (rVLM), a center that regulates sympathetic neural outflow. Tjen-A-Looi et al. [18] recorded single-cell neural activity in the rVLM during EA to evaluate point-specific responses of presympathetic neurons to stimulation of P56, LI4-L7, LI6-7, LI10-11, S36-37 and K1-B67 in cats [18]. Activity in the rVLM increased during stimulation of all acupoints but was significantly higher during stimulation of P5-6, located over the median nerve; LI10-11, located over the deep radial nerve and S36-37, situated over the deep peroneal nerve. The duration of the change in rVLM-evoked activity also varied with the acupoint, with P5-6 producing a significantly longer response. The point-specific actions resulting from stimulation of different acupoints in controlled laboratory trials confirm that needling different points on the body produces more than just placebo responses, given that placebo acupuncture is not associated with differential or acupoint-specific responses in anesthetized animals.

## Conclusion

Recent evidence shows that stimulation of different points on the body causes distinct responses in hemodynamic, fMRI and central neural electrophysiological responses. Brain imaging studies have shown that stimulation of different points elicits unique brain patterns that also are disease-specific. While fMRI studies show that different regions of the brain are activated, these studies do not show the pathways through which these changes occur. Laboratory studies of point specificity have demonstrated that stimulation of underlying neural pathways connecting variably to regions of the brain leads to differential physiological and clinical responses. However, because of our limited knowledge of the mechanisms underlying point specificity, further studies in this area are warranted.

## Competing interests

The authors declare that they have no competing interests.

## Authors' contributions

JCL conceived the article and coordinated its writing. EMC drafted the article. EMC and FJ analyzed past studies and gathered past articles for review. FJ and JCL edited the draft. EMC and FJ drafted responses to the reviewers, which were edited by JCL. All authors read and approved the final versions of the manuscript.
